# How to Decide Whether to Move Species Threatened by Climate Change

**DOI:** 10.1371/journal.pone.0075814

**Published:** 2013-10-16

**Authors:** Tracy M. Rout, Eve McDonald-Madden, Tara G. Martin, Nicola J. Mitchell, Hugh P. Possingham, Doug P. Armstrong

**Affiliations:** 1 School of Botany, University of Melbourne, Melbourne, Victoria, Australia; 2 School of Biological Sciences, The University of Queensland, Brisbane, Queensland, Australia; 3 Centre of Excellence for Environmental Decisions, The University of Queensland, Brisbane, Queensland, Australia; 4 Climate Adaptation Flagship, Commonwealth Scientific and Industrial Research Organisation, Brisbane, Queensland, Australia; 5 Centre for Evolutionary Biology The University of Western Australia, Perth, Western Australia, Australia; 6 Department of Mathematics and Physics, The University of Queensland, Brisbane, Queensland, Australia; 7 Wildlife Ecology Group, Massey University, Palmerston North, New Zealand; 8 Oceania Chair, International Union for the Conservation of Nature/Species Survival Commission Reintroduction Specialist Group, Abu Dhabi, United Arab Emirates; University of Kent, United Kingdom

## Abstract

Introducing species to areas outside their historical range to secure their future under climate change is a controversial strategy for preventing extinction. While the debate over the wisdom of this strategy continues, such introductions are already taking place. Previous frameworks for analysing the decision to introduce have lacked a quantifiable management objective and mathematically rigorous problem formulation. Here we develop the first rigorous quantitative framework for deciding whether or not a particular introduction should go ahead, which species to prioritize for introduction, and where and how to introduce them. It can also be used to compare introduction with alternative management actions, and to prioritise questions for future research. We apply the framework to a case study of tuatara (*Sphenodon punctatus*) in New Zealand. While simple and accessible, this framework can accommodate uncertainty in predictions and values. It provides essential support for the existing IUCN guidelines by presenting a quantitative process for better decision-making about conservation introductions.

## Introduction

Introducing individuals of a species to locations where the climate is predicted to be suitable in the future, but where the species has never occurred before, is a controversial adaptation action for combating the predicted impacts of climate change on biodiversity [1], [2], [3], [4], [5], [6], [7]. This action is a type of conservation introduction [8], and has been referred to as ‘managed relocation’, ‘assisted migration’ or ‘assisted colonization’ in recent debate about its implementation [3]. While discussion continues, conservation introductions motivated by climate change are already taking place [9], [10]. There are a number of critical decisions faced by those considering introductions as a potential climate adaptation strategy, and it is imperative that these decisions are aided by explicit, transparent and mathematically rigorous decision-making frameworks [11]. While such a framework has been used to determine the optimal timing of introductions motivated by climate change [12], several other key management questions remain unanswered.

The first, and most hotly debated, question is whether the benefit of introducing a species outside of its historical range in response to climate change is worth the financial costs and ecological risks [3]. Here the decision-maker must reflect not only on the potential collateral damage to the ecosystem at the introduction site, but also on the likelihood that the introduction will be successful, the benefit to the species of concern from a successful introduction, and the relative importance placed on this species by the decision-maker. Two recent studies [1], [2] develop frameworks for addressing this question, but neither do so within the tenets of decision science: both lack a quantifiable management objective and mathematically rigorous problem formulation.

As well as this key question of whether or not to go ahead with the introduction of an individual species, there are a number of related questions that need to be addressed for good decision-making. We must decide how best to implement an introduction, and where the introduction will occur. Further, these introductions incur a financial cost, and we will never have enough money to translocate every candidate species under threat, so we must consider budgetary constraints when prioritizing how and where to introduce species, and also when prioritizing among candidate species for introduction. Here we provide the first quantitative decision framework that can be used to address any, or all of, these questions. By formulating these questions in a rigorous way, we identify which predictions are necessary for making an informed decision. This framework should be applied pre-emptively, to direct future ecological research towards answering the questions most relevant to management decisions.

## Materials and Methods

### The decision framework

We propose a decision framework that can assist in deciding whether or not to implement a specific introduction, and also in choosing between candidate species (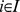) for introduction, different strategies (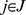) for introducing each species, and different locations (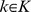) to introduce each species. This framework is targeted towards a single decision-maker, for example, a government agency or non-governmental conservation organisation, whose aim is to save species from extinction and who has identified one or more species likely to go extinct in their current range as it becomes unsuitable due to climate change, and for which an introduction outside their historical range is a possible option. The decision-maker wishes to assess whether the benefits of an introduction outweigh the risks for those species, and to prioritise different introduction options.

**Figure 1:**
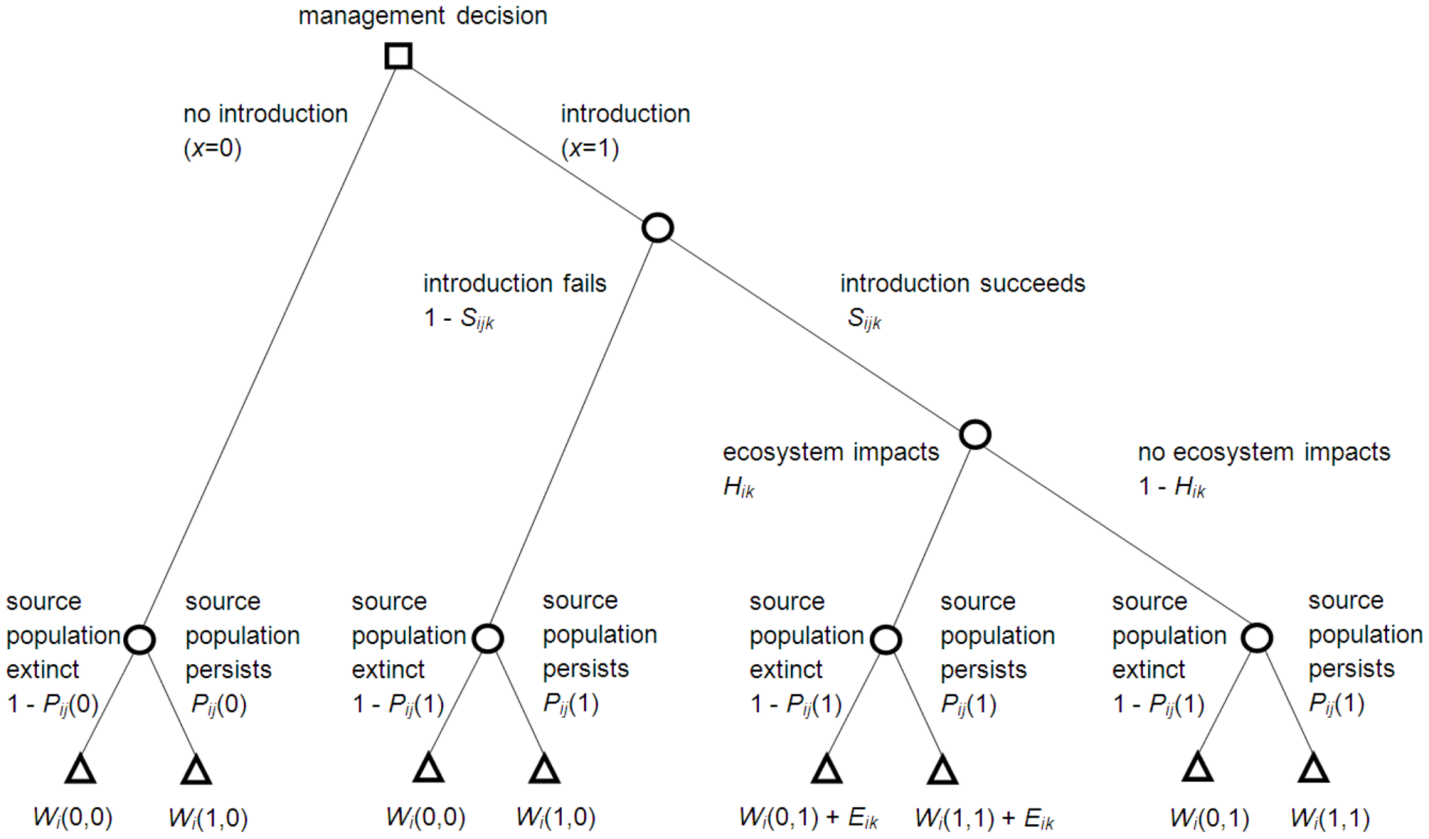
A decision tree describing the conservation introduction problem. Squares represent decision nodes, circles are stochastic events, and triangles are outcomes. The expected value of each choice is calculated by multiplying down the branches of the tree to obtain the probability each outcome will occur, and summing across the possible outcomes under each choice. For example, if we choose not to introduce (*x* = 0), there is a probability 1– *P_ij_*(0) that the source population will go extinct (outcome *W_i_*(0,0)), and probability *P_ij_*(0) that the source population will persist (outcome *W_i_*(1,0)). The expected value of choosing not to introduce is the sum of these two expected values, i.e. (1– *P_ij_*(0))*W_i_*(0,0) + *P_ij_*(0)*W_i_*(1,0).

Our management decision for a given species *i*, strategy *j*, and location *k*, is to either implement introduction (*x* = 1) or not implement introduction (*x* = 0). The decision tree for this choice is described in Figure 1. This decision tree assumes events occur in a certain order, from the top to the bottom of the tree. This means, for example, that we assume the extinction of the source population, if it happens, occurs after the introduction takes place (Figure 1).

If introduction is chosen, it may or may not be successful. We assume the probability of success, *S_ijk_*, depends on the species *i*, strategy *j*, and location *k*. If an introduced population is successfully established, there is a probability *H_ik_* that it will have an undesirable impact on other species or on ecosystem function at location *k*. Let *E_ik_* be the predicted impact (weighted by its relative importance compared to species persistence outcomes – see below), should the introduced species *i* cause undesirable impacts at location *k*. We assume the species cannot colonize these locations without being introduced, although a probability of natural colonization can easily be incorporated (see below for a discussion).

Let *P_ij_*(*x*) be probability that the source population of species *i* will persist, assuming we take action *x*, using strategy *j* (if *x* = 1). Introduction may involve the removal of individuals or propagules from the source population, which could have a negative effect on its persistence, i.e. *P_ij_*(1) ≤ *P_ij_*(0). Implementing an introduction could also impact on the source population if resources are diverted from other management actions that support this population.

We care about the persistence of the source population, as well as the new population at the introduction site. To describe this, let *y* be the state of the source population, where *y* = 1 if extant and 0 otherwise, and let *z* be the state of the introduced population, where *z* = 1 if extant and 0 otherwise. Let *W_i_*(*y, z*) be the relative value of species *i* (weighted against the value of predicted impacts *E_ik_* – see below), given the final state of each population. For example, *W_i_*(1, 0) is the relative value of species *i* persisting only at the source location, while *W_i_*(0, 1) is the value of species *i* persisting only at the introduction site. We may consider these populations to be valued equally, i.e. *W_i_*(1, 0)  =  *W_i_*(0, 1), or we may prefer the species to persist at the source location if possible, i.e. *W_i_*(1, 0) > *W_i_*(0, 1). These weights reflect value judgements about the possible outcomes of decisions, and should be set by decision-makers in consultation with experts and stakeholders. Value judgements are simply a statement of what the decision-maker thinks is important in the context of the choice to be made, and are a necessary component of any decision [13]. Eliciting value judgements independently from scientific predictions allows these judgements to be viewed explicitly, and minimises the opportunity for values to bias predictions of future outcomes under different actions [13].

There are no set units for quantifying the weighted value of the final state of species *i*, *W_i_*(*y, z*), or the weighted value of predicted impacts at the destination site, *E_ik_*. However, because these values are combined to calculate the expected benefit, they must be commensurable. One way to ensure this is to elicit these values on the same constructed scale, e.g., rating the desirability of each outcome on a scale from −1 to 1, where positive numbers represent outcomes the decision-maker considers desirable, and negative numbers outcomes the decision-maker considers undesirable [14]. Alternatively, these values could be elicited in different, more natural units, such as the number of species at the introduction site predicted to be negatively affected by the introduction. They can then be weighted against each other using multi-criteria decision analysis methods (e.g., swing weighting), which formalize the decision-maker's preferences with regard to conflicting objectives [13].

These preferences could be formed by taking into account the range of views of relevant stakeholders, who might hold very different ideas about the values of *W_i_*(*y*,*z*) and *E_ik_*. Assigning weights such as these is an emotionally and cognitively difficult task [13], but one that is routinely performed in multi-criteria decision analysis. Methods have therefore been developed to reduce this burden, and to ensure elicited weights are a true reflection of the decision-maker's or stakeholder's preferences [13].

The net expected benefit of introducing species *i* to location *k* using strategy *j* is the expected value of this introduction (*x* = 1) minus the expected value of doing nothing (*x* = 0). This can be derived from the decision tree (Figure 1) to be:
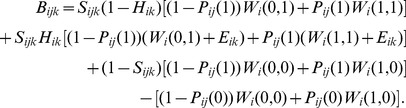
(1)


To accommodate the possibility that ecosystem impacts may be positive or negative, we have added the predicted impact *E_ik_*, such that impacts viewed as undesirable by the decision-maker must be expressed as a negative number. For an individual species, strategy, and location, it is worthwhile proceeding with the introduction when *B_ijk_* is positive, i.e., when introducing is better than doing nothing.

A decision-maker who seeks the best expected outcome can be described as being risk neutral [13]. A risk-averse decision-maker would be more concerned with minimizing negative outcomes. The risk-averse decision-maker can also be accommodated in our decision tree, for example, by choosing the decision with the smallest worst-case outcome, or with the lowest chance of obtaining a negative outcome.

### Multiple species, locations, and strategies

In situations where a decision-maker is faced with many species, locations or introduction strategies to choose from, these options could be prioritized by simply choosing those with the largest expected benefit. However, conservation resources are always limited, and to get the biggest conservation benefit with the resources available, we must take into account the cost of different options.

Cost-effectiveness analysis is a type of economic analysis comparing the relative outcomes and costs of different options. It differs from cost-benefit analysis in that the outcomes of decisions (in this case the net benefit of an introduction) do not need to be monetized. Cost-effectiveness analysis is a useful approach for conservation prioritization, because it can explicitly incorporate financial considerations while avoiding the ethical and practical dilemmas associated with putting a monetary value on species or ecosystems [15]. Cost-effectiveness analysis has been used to prioritise threatened species recovery projects [15], [16], actions to mitigate threats to biodiversity [17], and landscape-scale environmental projects [18].

Options are evaluated by dividing their expected benefit (in any unit) by their cost, to obtain a cost-effectiveness ratio. This metric can then be used to prioritize options. If the decision-maker seeks to maximize the benefit that can be achieved with a fixed management budget, they should fund the most-cost effective option first, and work down the list until their budget is exhausted [16], [17]. This simple approach approximates an optimal allocation of resources, as long as the budget is reasonably large compared to the cost of each option [19].

When evaluating options across multiple species, the benefit of introducing a species incorporates the relative value of that species compared with the other candidate species for introduction. This is expressed in the weight *W_i_*. All species could be valued equally, or they could be weighted based on factors such as cultural significance, ecological function, economic importance, taxonomic distinctiveness, or a combination of several factors [16].

For introductions, a cost-effectiveness analysis can be applied as a sequence of decisions:

1. For a specific species, *i*, for one location, *k*, use equation 1 above to find the expected benefit of each translocation strategy, *B_ijk_*.
2. Then find for a specific species, *i*, in that location, *k*, which strategy is the most cost effective, for all strategies *j*, where the benefits and risks outweigh those of doing nothing. Here we divide each strategy's expected benefit, *B_ijk_,* by the cost of its implementation, *C_ijk_,* and find the most cost effective strategy j*.
3. Repeat step 1 and 2 for each location *k*, for an individual species *i*, and find the most cost effective location for introduction of that species.
4. Repeat 1 to 3 for each species.
5. To trade off between all species *I*, we can rank all most cost effective locations for each species and spend our budget from the top down until our budget is exhausted.

See Supporting Information (File S1 with Tables S1, S2, S3, and S4) for an illustrative example of how this procedure is applied.

This prioritization framework can also be used to compare introduction options with alternative plausible management actions for mitigating the effects of climate change, such as managing threats to the species within its current range, managing the species *ex situ*, or managing connectivity to facilitate natural colonization.

### Estimating model parameters

To make a considered decision about an introduction, we must make predictions about the likelihood that the source population will go extinct, the likelihood that the planned introduction will be successful, and the likely ecosystem impacts at the introduction site. Although analytical methods for making these predictions are rapidly evolving [20], these methods require time, effort, expertise, and most importantly, data. For any candidate species, it is therefore unlikely that all these predictions will be readily available. Rather than being a barrier to rigorous decision-making, this lack of information should be seen as a motivator for using such a framework. By first implementing the framework with currently available information, the key parameters affecting a decision to introduce can be identified. Future research can then be directed towards estimating those parameters for which more information is needed to be able to make a good decision.

Most predictions of species persistence under climate change are based on empirical niche models [21], also known as climate-envelope models. While these models are useful for predicting the likely shift in distribution experienced by the species as a result of climate change, they do not account for the species' sensitivity to climate change, nor its adaptive capacity [22]. However, moves to integrate climate envelope models with stochastic population models provide a way to couple some of this information [23], [24]. Furthermore, their outputs can be easily translated into extinction risks. Integrating different types of available information, such as empirical and observational data, and mechanistic population and ecophysiological models, will give a more nuanced and robust prediction of the effect of climate change on a species [22].

Stochastic population models can also be used to predict the success of introductions and other translocations, and are now a standard approach for planning and evaluating the success of translocations [25]. Furthermore, numerous reviews of previous translocation programmes [26] can provide a means for predicting translocation success based on the characteristics of the species (e.g., mammals versus birds) or the translocation programme (e.g., the number of individuals released and whether supplementary food is provided). Combined with forecasts of future climatic suitability to identify appropriate sites for introductions, these methods can provide a basis for predicting the success of conservation introductions in response to climate change.

Predicting which introduced plants and animals will become invasive and the type of impacts they are likely to have is understandably a question of great interest to government agencies involved in import risk assessment. For this reason there are well-established methods for making these predictions, with data from previous introductions used in trait-based models to assess the threat posed by new species. For example, the Australian Weed Risk Assessment system is based on information about a species' weed status in other parts of the world, its biological attributes, and its climate and environmental preferences [27]. While it has been used to assess plant importations into Australia since 1997, the system has also been adapted and tested in many countries and provinces around the world [28]. Trait-based models of invasiveness have also been developed for animal species [29]. Recent research suggests that generalisations can also be made about the susceptibility of an ecosystem to plant invasion [30], and the magnitude and type of impact that is likely based on the characteristics of the system and the introduced plant species [31].

For some species there may be neither the data nor the time to make these predictions analytically. In these cases, predictions can be elicited from those with knowledge and experience of the relevant species and ecosystems. A variety of methods have been developed for ensuring information elicited from experts is as reliable as possible, is unbiased, and accurately represents uncertainty [32]. Expert judgement can also be used in combination with empirical data to increase the power of a model when data are scarce [14].

Regardless of their source, these predictions will be uncertain. Value judgements about the population weights and the potential impact at the site of introduction may also be uncertain, particularly if they represent the combined judgements of different stakeholders. To quantify uncertainty, parameters can be specified as probability distributions describing the likelihood of the parameter taking on different values. These can be used to generate a distribution for the expected benefits of each management option (Figure S1). With uncertainty in parameter estimates, it may be appropriate to reformulate the objective to account for the decision-maker's attitude to risk, as mentioned above. For example, instead of choosing the option with the highest expected benefit (the mean value of the distribution of expected benefits), we may prefer the option with the largest chance of a positive benefit, or the smallest chance of a negative benefit, given the specified uncertainty in parameter estimates. Sensitivity analyses can also be used to examine the effect of changes in parameter values on the best decision and its likely outcome. Sensitivity analyses or formal value of information analyses [33] can identify parameters for which more information is needed to make a good decision, thus prioritizing areas for future research.

## Results

### When should we introduce?

Using equation 1 we derived some general circumstances for when an introduction should go ahead. A simple assumption about the value of population outcomes is that the decision-maker cares only whether the species persists, giving no value to extinction (*W_i_*(0,0)  = 0), and equal value to the scenarios where one or both populations persist (*W_i_*(1,0)  =  *W_i_*(0,1)  =  *W_i_*(1,1)). This assumption will not always be the case and these relative values should be carefully considered for each species. However, if the assumption holds, the net expected benefit of introduction is:
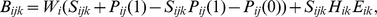
where *W_i_* is the value of the species persistence. It will be worthwhile proceeding with the introduction if this benefit is positive, which occurs when



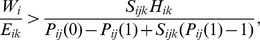
where *W_i_*/*E_ik_* expresses the importance of the focal species relative to its potential impact at the introduction site. If the introduction has no detrimental effect on the source population (*P_ij_*(1)  =  *P_ij_*(0)), then this simplifies to:



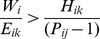



In this case the probability of success does not influence the decision of whether to introduce, but it does determine the size of the expected benefit.

On the other hand, if the introduction will impact on the source population but not on the ecosystem at the introduction site (*E_ik_* = 0), then the introduction should proceed if its probability of success is greater than the relative cost to the source population, i.e.
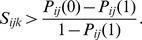



This means that if there is no chance of the source population going extinct, then it will never be optimal to introduce.

If the source population is certain to go extinct regardless of what we do (*P_ij_*(0)  =  *P_ij_*(1)  = 0), then the benefit of introduction is simply:
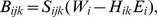
and the introduction should proceed if we value the focal species more than its expected impact at the introduction site, i.e., *W_i_* > –*H_ik_E_i_*.

### Case study: an introduction of tuatara

To illustrate how the framework might be applied to a candidate introduction, we used a case study of tuatara (*Sphenodon punctatus*), a threatened species of reptile endemic to New Zealand. Tuatara were once widespread throughout New Zealand, but became restricted to around 30 offshore islands northeast of the North Island and in the Cook Strait following the introduction of mammalian predators [34]. Tuatara have temperature-dependent sex determination, with higher incubation temperatures giving rise to males and lower temperatures to females [35]. Male-biased sex ratios have already been observed in the North Brother Island population, and are expected to increase as temperatures increase, putting this population at risk of extinction [36], [37]. Successful introductions of tuatara within the same ecoregion have already occurred, with introductions outside of current ranges planned for the future [34].

For this illustration, we consider introducing tuatara from the well-studied and genetically distinct North Brother Island population to a single location: a hypothetical mainland sanctuary on New Zealand's South Island (with a higher latitude and cooler climate). Although fossil records show tuatara were historically present on the South Island, there is a lack of information about their presence at a local scale and it is unknown whether extirpated populations were the same species as the current island populations [34]. For these reasons this translocation would be considered an introduction rather than a reintroduction [34].

Temperature has a direct effect on the demographics of tuatara, therefore we used a previously published population viability model that ran over 2000 years [36] to predict the effect of climate change on the North Brother Island population. The long life expectancy of tuatara (approximately 100 years) led us to consider a revised timeframe of 150 years into the future for our predictions. Under a maximum climate change scenario of 3–4°C warming, where sex ratios increase linearly from 50% in 2002 and stabilise at 100% males by 2085 [36], [37], the probability that the current population of 550 individuals will persist (with both males and females) in 150 years is *P_ij_*(0)  = 0.43 (averaged over 500 simulations). Our proposed strategy of removing 50 individuals for translocation has little effect on viability, with *P_ij_*(1)  = 0.42.

**Figure 2:**
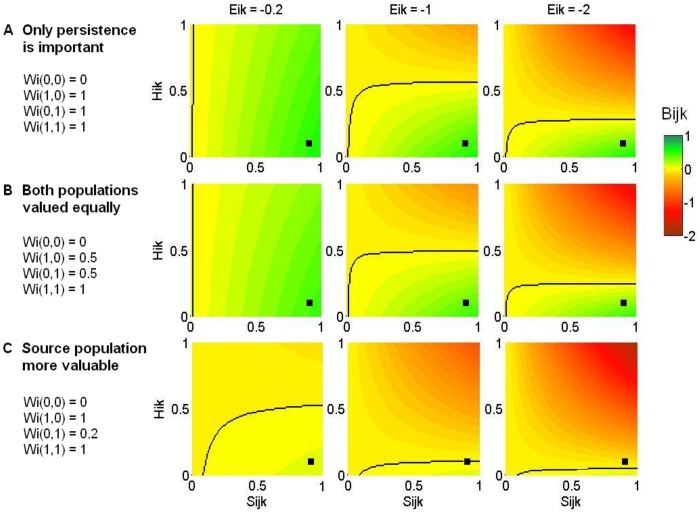
The expected benefit of introducing tuatara (*B_ijk_*). Shown for different values of: the probability that the introduction will be successful, *S_ijk_* (x-axes), the probability of an impact on the ecosystem at the new site *H_ik_* (y-axes), the weighted population outcomes *W_i_* (rows), and the weighted magnitude of the ecosystem impact *E_ik_* (columns). Black lines mark the change in the best management decision: introduce when the benefit is positive (right/below) and do nothing when the benefit is negative (left/above). Black squares mark best estimates for our tuatara introduction.

If we were considering a real candidate translocation site for tuatara, we could use the same population viability model modified according to location-specific demographic parameters (e.g. hatchling sex ratios, reproductive rates) to estimate the long-term viability of an introduced population at that site. However, because our introduction site in this example is hypothetical, we used an estimate based only on the high success rate of previous tuatara introductions (*S_ijk_*  = 0.9) [34]. We also conducted a sensitivity analysis to look at the effect of changes in this parameter on the best decision for tuatara, including best-case and worst-case scenarios (Figure 2). Decreasing the probability of introduction success from our best estimate (black squares) decreases the magnitude of the expected benefit obtained, whether positive (green) or negative (red). However, changes in this probability have little effect on the decision to introduce (black lines), unless the probability is very low.

We made an estimate of the probability of ecosystem impacts at our hypothetical introduction site based on previous tuatara introductions, which have had no discernible negative impacts on the ecosystems at those sites (*H_ik_*  = 0.1, black squares in Figure 2) [34]. Our sensitivity analysis shows that the best decision can be insensitive to the probability of ecosystem impacts, if the magnitude of those impacts is predicted to be small (Figure 2, left column). However, the larger the predicted impacts, the smaller the threshold probability at which becomes better to not introduce (Figure 2, middle and right columns).

Tuatara are culturally significant for both European and Māori stakeholders [34]. We did not estimate relative values for tuatara, but instead examined results across a range of different possibilities. Given our best estimates of other parameters, the relative value placed on the source and introduced populations can affect the optimal decisions (black squares, Figure 2). If only the persistence of the species matters, not the number of populations, it is optimal to introduce tuatara for the range of ecosystem impacts investigated (Figure 2A). These results change only slightly if both the source and introduced populations are valued equally and additively (Figure 2B). However, in a scenario where an introduced population is considered less valuable than the source, the benefits of introduction are greatly reduced and it is only optimal to introduce if ecosystem impacts are predicted to be small (Figure 2C).

## Discussion

We have focused here on conservation introductions in response to climate change, which are only a subset of cases where a species is introduced to an area outside of its historical range for conservation purposes. Many such introductions have already taken place, most commonly to establish populations isolated from introduced predators, but also to re-establish the functional roles played by extinct forms, most frequently using close taxonomic relatives [38]. Indeed in our tuatara example, conservation introductions are considered not only to mitigate the threat of climate change, but also to restore ecosystems to a state similar to that before mammalian invasions [34]. Our framework can easily be adapted for this alternative motivation,(by specifying a positive ecosystem impact *Ei_k_*) or applied to decisions about conservation introductions generally. The framework is intended to support the revised “IUCN guidelines for re-introductions and other conservation translocations”, which explicitly calls for structured decision-making frameworks for conservation introductions [39].

When applying this framework, additional events or outcomes relevant to the focal species can be incorporated into the decision tree. For example, it may be pertinent to include the possibility that the species will colonize the introduction site naturally (see Figure S2 for a decision tree with this possibility included). Expanding the decision tree means judgements must be made about where the new event fits within the timeline of events, and how the outcomes of the new event will be valued. For example, we must decide how to weight the potential undesirable impacts of a natural colonization (see Figure S2). As with any mathematical formulation of an environmental problem, there is a trade-off between realism and utility, and the more complex the decision tree, the less accessible it will be as a decision support tool. It is therefore important to include only the events and outcomes that are most relevant to the decision being made.

Having said this, our framework makes several simplifications that are worth noting. We do not explicitly consider how the extinction of the source population will affect other species at that site, although a relative measure of the importance of the source population can be expressed in the weightings *W*(*y*,*z*). Using a decision tree means extinction and population establishment are treated as discrete events occurring in a specified order, when in reality these events may occur simultaneously over many years. These details are considered in the dynamic model McDonald-Madden et al. [12] used to determine the optimal timing of introductions in response to climate change. These two decision frameworks could be applied in a complementary way, using our decision tree to first assess whether an introduction should go ahead, and then the detailed dynamic model to determine the best timing for that introduction.

Decisions regarding conservation under climate change will be fraught with uncertainty, complexity, and conflicting values. It is in precisely these situations that a logical approach to decision-making is most needed [11]. Although, our framework is simple, it is general, transparent and adaptable, and can be informed by quantitative models and data analyses and/or rigorously elicited expert judgements. Regardless of the amount or type of information available, decisions based on a transparent framework are preferable to the alternative, namely opaque, *ad hoc* decision-making.

## Supporting Information

Figure S1 **Probability distribution for the simulated benefit (** ***B_ijk_*** **) of a hypothetical conservation introduction, using the decision tree without natural colonization.**
(DOCX)

Figure S2 **A decision tree that considers the probability that the source population will persist(** ***P_ij_*** **), the probability that a new population is established at a proposed location through successful introduction (** ***S_ijk_*** **) or through natural colonization (** ***N_ik_*** **), and the probability that successful establishment at the new site will impact on the ecosystem there (** ***H_ik_*** **).**
(DOCX)

File S1 **Supplementary Methods: An illustrative example of species prioritization.**
(DOCX)
