# Supplementary material for: How to Decide Whether to Move Species Threatened by Climate Change
Source: PLoS One. 2013 Oct 16;8(10):e75814. doi: 10.1371/journal.pone.0075814 (PMC3797766; doi:10.1371/journal.pone.0075814)
Supplement: Figure S1 — Probability distribution for the simulated benefit ( Bijk ) of a hypothetical conservation introduction, using the decision tree without natural colonization. (DOCX) [file pone.0075814.s001.docx]

**Figure S1.**


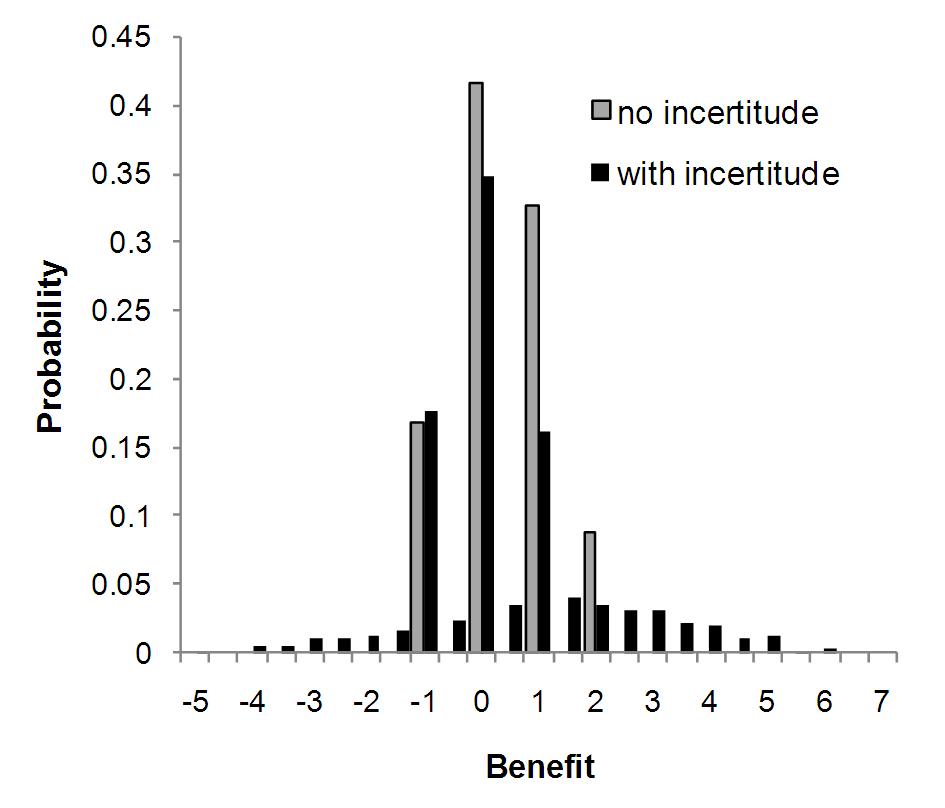


Probability distribution for the simulated benefit (*B_ijk_*) of a hypothetical conservation introduction, using the decision tree without natural colonization. Grey bars show the distribution generated when best estimates of the parameters are used, and the outcome of stochastic events is simulated through 1000 iterations. The black bars show distribution of benefits generated when parameters are randomly drawn from distributions based on their estimates and standard errors. The probabilities *S_ijk_* and *P_ij_*(*x*) are logit-normally distributed and the expected impact at the introduction site is a normally distributed continuous random variable, rather than a fixed impact *E_ik_* occurring with probability *H_ik_*. The estimates (and standards errors) are: The values of population outcomes are considered fixed at *W_i_*(0,0) = -1, *W_i_*(1,0) = 0, *W_i_*(0,1) = 0, *W_i_*(1,1) = 1, but could also be treated as random variables.
