# Supplementary material for: How to Decide Whether to Move Species Threatened by Climate Change
Source: PLoS One. 2013 Oct 16;8(10):e75814. doi: 10.1371/journal.pone.0075814 (PMC3797766; doi:10.1371/journal.pone.0075814)
Supplement: Figure S2 — A decision tree that considers the probability that the source population will persist( Pij ), the probability that a new population is established at a proposed location through successful introduction ( Sijk ) or through natural colonization ( Nik ), and the probability that successful establishment at the new site will impact on the ecosystem there ( Hik ). (DOCX) [file pone.0075814.s002.docx]

**Figure S2.**


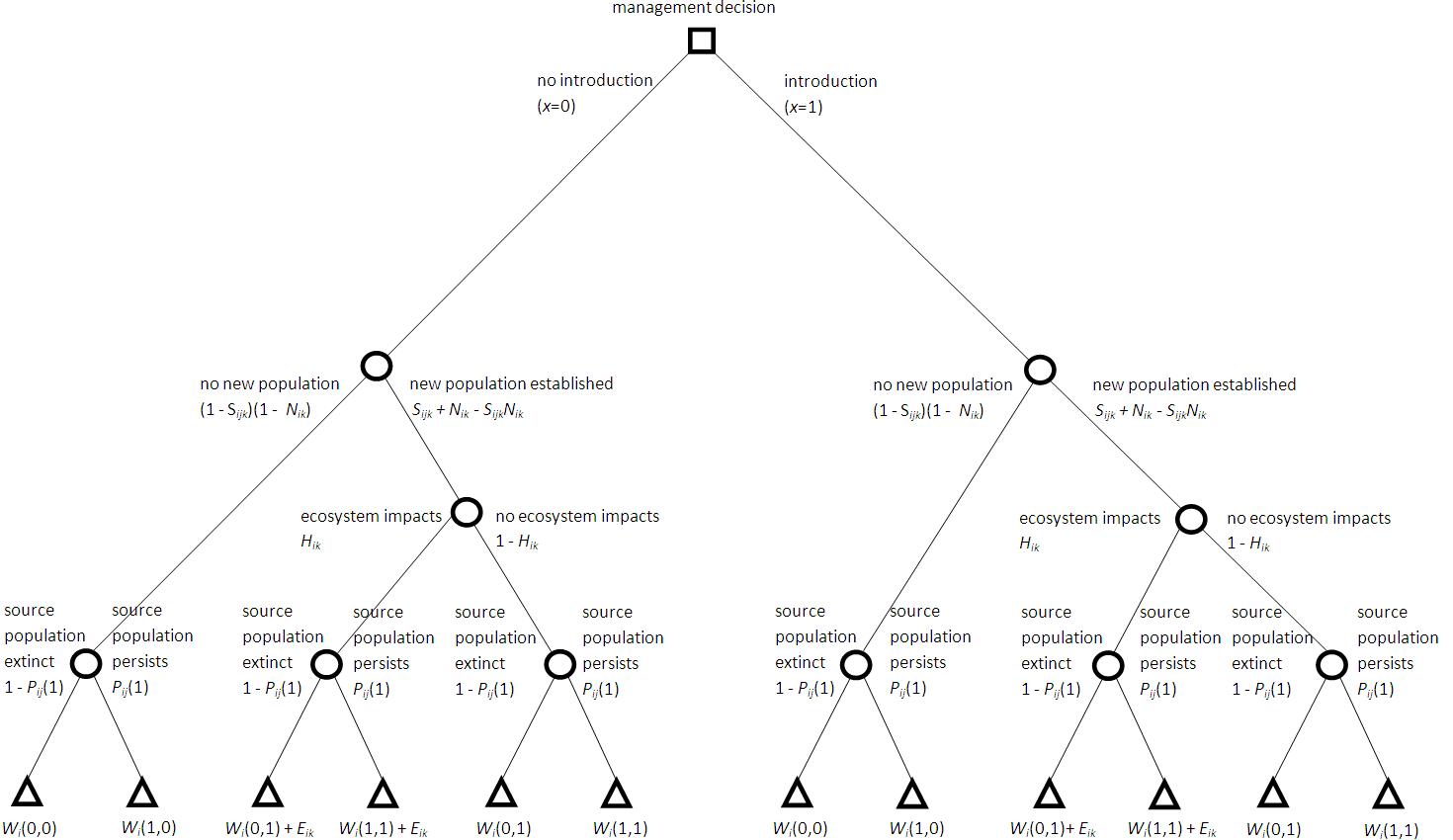


A decision tree that considers the probability that the source population will persist(*P_ij_*), the probability that a new population is established at a proposed location through successful introduction (*S_ijk_*) or through natural colonization (*N_ik_*), and the probability that successful establishment at the new site will impact on the ecosystem there (*H_ik_*). This decision tree treats all ecosystem impacts equally, whether they are the result of a successful introduction or a natural colonization. The square represents the management decision, circles are stochastic events, and triangles are outcomes. The utility of an outcome depends on values assigned to populations of the species (*W_i_*) in relation to negative impacts on the ecosystem (*E_ik_*). The expected utility of each choice (*x* = 0, *x* = 1) is calculated by multiplying down the branches to obtain the probability each outcome occurring, and summing across the possible outcomes.
