# Supplementary material for: How to Decide Whether to Move Species Threatened by Climate Change
Source: PLoS One. 2013 Oct 16;8(10):e75814. doi: 10.1371/journal.pone.0075814 (PMC3797766; doi:10.1371/journal.pone.0075814)
Supplement: File S1 — Supplementary Methods: An illustrative example of species prioritization. (DOCX) [file pone.0075814.s003.docx]

**File S1: Supplementary Methods**

**An illustrative example of species prioritization.**

Suppose we must prioritize the introductions of three (imaginary) species threatened by climate change: the black-spotted butterfly, the alpine pygmy mouse, and the northern blue pine. Each species has two possible locations and two possible strategies for introduction. We consider these three species to be equally important, and for each species we do not mind how many populations persist as long as the species is extant (these values are reflected in the population outcome weights *W*(*y_o_*, *y_d_*)). For each species we compare simple strategies of introducing few or many individuals. Note that these are not real species - life history information is mentioned for illustrative purposes only.

The black-spotted butterfly (Table S1) relies on a host plant, and two candidate sites for introduction occupied by this plant have been identified. The species is predicted to compete with a similar butterfly species at one of these sites. Removing individuals from the source population will substantially affect its viability, and early trials to capture and release the species have been of mixed success.

The alpine pygmy mouse (Table S2) persists in small numbers at one alpine site. Captive populations have already been established and can be used to supply individuals for the introductions, so there will be no impact of introduction on the source population. The species is unlikely to impact on the ecosystem at either candidate introduction site. Introductions have a moderate chance of being successful, but will be expensive.

The northern blue pine (Table S3) can be propagated by seeds, so the introduction will have no effect on the source population. Introductions will be cheap, and are likely to be successful. However, the species is also likely to have a negative impact on the ecosystems at the introduction sites.

We first focus on the black spotted butterfly (Table S1). Following the prioritization procedure outlined in the paper, we use equation 1 to first calculate the expected benefit of each strategy at location 1. Strategy 1 is less likely to be successful than strategy 2 in establishing an introduced population, but it also has less of an impact on the source population and is less costly to implement. Both strategies have a positive benefit, so an introduction with either strategy would be worthwhile, that is, the expected benefit to the black-spotted butterfly outweighs the risks of an undesirable impact at this location. Strategy 2 has a larger expected benefit than strategy 1, and is also the most cost-effective at this location (*j** = 1).

Performing the same calculations for location 2, we find that because of the predicted negative impact at this location, both strategies have a negative benefit. Introduction at this location should not go ahead, because the risks of an undesirable impact are greater than the expected benefit to the species. We now know that introducing the black-spotted butterfly at the best location (*k** = 1) using the best strategy (*j** = 1) has a cost-effectiveness ratio of 2.55.

We then repeat these calculations for the alpine pygmy mouse and the northern blue pine (Tables S2 and S3). We can again identify the most cost-effective strategies of introduction at each location, and subsequently find the most cost-effective location for introduction of each species. The best strategies and locations for each species are marked with *.

Compiling this information (Table S4) allows us to prioritize investment in these three species. The first priority (with the largest cost-effectiveness ratio) is the alpine pygmy mouse, followed by the black-spotted butterfly, and the northern blue pine. With $370,000, we could fund the best introduction option for all three species. If our budget is less than this, we should start by funding the top priority first, and move down the list until the budget is exhausted.

**Table S1**: Best estimate parameters for the black-spotted butterfly (*i* = 1)

| Parameter | Best estimate | | | |
| --- | --- | --- | --- | --- |
| Probability source population will persist in 20 years if there is no introduction, *P_ij_*(*x*=0) | 0.3 | | | |
| Weighted value of population outcomes,  *W_i_*(*y_o,_ y_d_*) | *W_1_*(0,0) = 0, *W_1_*(1,0) = 1, *W_1_*(0,1) = 1, *W_1_*(1,1) = 1 | | | |
|  | Location 1 (*k* = 1) | | Location 2 (*k* = 2) | |
| Probability of undesirable impacts at introduction location, *H_ik_* | 0.1 | | 0.6 | |
| Weighted magnitude of undesirable impacts at introduction location, *E_ik_* | -1 | | -1 | |
|  | Strategy 1(*j* = 1)  move few individuals | Strategy 2 (*j* = 2)  move many individuals | Strategy 1 (*j* = 1)  move few individuals | Strategy 2 (*j* = 2)  move many individuals |
| Probability of successful introduction, *S_ijk_* | 0.4 | 0.6 | 0.4 | 0.6 |
| Probability source population will persist, *P_ij_*(*x*=1) | 0.2 | 0.1 | 0.2 | 0.1 |
| Expected benefit of introducing at location *k* using strategy *j*, *B_ijk_* | 0.18 | 0.28 | -0.02 | -0.02 |
| Cost of introduction, *C_ijk_* | $80,000 | $110,000 | $80,000 | $110,000 |
| Cost-effectiveness ratio, *B_ijk_*/*C_ijk_* (/$10^6^) | 2.25 | 2.55* | -0.25 | -0.18 |

**Table S2**: Best estimate parameters for the alpine pygmy mouse (*i* = 2)

| Parameter | Best estimate | | | |
| --- | --- | --- | --- | --- |
| Probability source population will persist if there is no introduction, *P_ij_*(*x*=0) | 0.1 | | | |
| Weighted value of population outcomes,  *W_i_*(*y_o,_ y_d_*) | *W_2_*(0,0) = 0, *W_2_*(1,0) = 1, *W_2_*(0,1) = 1, *W_2_*(1,1) = 1 | | | |
|  | Location 1 (*k* = 1) | | Location 2 (*k* = 2) | |
| Probability of undesirable impacts at introduction location, *H_ik_* | 0.05 | | 0.05 | |
| Weighted magnitude of undesirable impacts at introduction location, *E_ik_* | -1 | | -1 | |
|  | Strategy 1 (*j* = 1) | Strategy 2 (*j* = 2) | Strategy 1 (*j* = 1) | Strategy 2 (*j* = 2) |
| Probability of successful introduction, *S_ijk_* | 0.4 | 0.6 | 0.5 | 0.7 |
| Probability source population will persist, *P_ij_*(*x*=1) | 0.1 | 0.1 | 0.1 | 0.1 |
| Expected benefit of introducing at location *k* using strategy *j*, *B_ijk_* | 0.34 | 0.51 | 0.425 | 0.595 |
| Cost of introduction, *C_ijk_* | $150,000 | $200,000 | $150,000 | $200,000 |
| Cost-effectiveness ratio, *B_ijk_*/*C_ijk_* (/$10^6^) | 2.27 | 2.55 | 2.83 | 2.98* |

**Table S3:** Best estimate parameters for the northern blue pine (*i* = 3)

| Parameter | Best estimate | | | |
| --- | --- | --- | --- | --- |
| Probability source population will persist if there is no introduction, *P_ij_*(*x*=0) | 0.4 | | | |
| Weighted value of population outcomes,  *W_i_*(*y_o,_ y_d_*) | *W_1_*(0,0) = 0, *W_1_*(1,0) = 1, *W_1_*(0,1) = 1, *W_1_*(1,1) = 1 | | | |
|  | Location 1 (*k* = 1) | | Location 2 (*k* = 2) | |
| Probability of undesirable impacts at introduction location, *H_ik_* | 0.7 | | 0.55 | |
| Weighted magnitude of undesirable impacts at introduction location, *E_ik_* | -1 | | -1 | |
|  | Strategy 1 (*j* = 1) | Strategy 2 (*j* = 2) | Strategy 1 (*j* = 1) | Strategy 2 (*j* = 2) |
| Probability of successful introduction, *S_ijk_* | 0.75 | 0.85 | 0.7 | 0.8 |
| Probability source population will persist, *P_ij_*(*x*=1) | 0.4 | 0.4 | 0.4 | 0.4 |
| Expected benefit of introducing at location *k* using strategy *j*, *B_ijk_* | -0.075 | -0.085 | 0.035 | 0.04 |
| Cost of introduction, *C_ijk_* | $60,000 | $70,000 | $60,000 | $70,000 |
| Cost-effectiveness ratio, *B_ijk_*/*C_ijk_* (/$10^6^) | -1.25 | -1.21 | 0.58* | 0.57 |

**Table S4:** The most cost-effective options for introduction of each species

|  | Black-spotted butterfly | Alpine pygmy mouse | Northern blue pine |
| --- | --- | --- | --- |
| Most cost effective location | *k** = 1 | *k** = 2 | *k** = 2 |
| Most cost effective strategy at this location | *j** = 2 | *j** = 2 | *j** = 1 |
| Cost | $110,000 | $200,000 | $60,000 |
| Cost-effectiveness ratio | 2.55 | 2.98 | 0.58 |
